# P-54. High Dose Intramuscular Influenza Vaccine in Solid Organ Transplant Patients. A Systematic Review and Meta-analysis

**DOI:** 10.1093/ofid/ofae631.261

**Published:** 2025-01-29

**Authors:** Reza Rahimi Shahmirzadi, Meisam Abdar Esfahani, Rachel Couban, Sarah Cairns, Sameer Elsayed, Michael Silverman

**Affiliations:** Western University, London, Ontario, Canada; Western University, London, Ontario, Canada; McMaster University, Hamilton, Ontario, Canada; McMaster University, Hamilton, Ontario, Canada; Western University, London, Ontario, Canada; Western University, Lawson Health Research Institute., London, Ontario, Canada

## Abstract

**Background:**

Seasonal influenza can cause serious illness and even death, especially in immunocompromised people such as solid organ transplant recipients (SOT). Early results from some studies show that high dose (HD) intramuscular (IM) vaccine might help boost the body's immune response in transplant patients. Given the uncertain benefits and harms associated with various IM vaccine doses a synthetized appraisal of the evidence is warranted.

**Figure 1:**
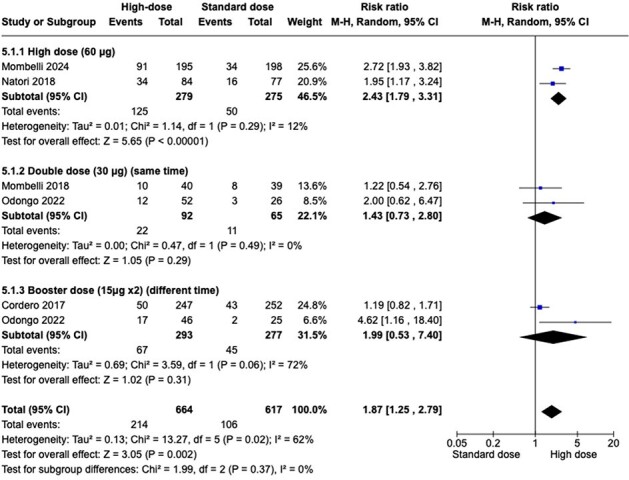
Seroconversion rate in Influenza A(H1N1) (SC H1N1) Forest plot

**Methods:**

Randomized controlled trials of adults who underwent SOT and received IM influenza vaccine and compared , IM influenza HD vaccine with standard dose. The outcomes of interests are seroconversion rate (SCR) for H1N1,H3N2, and graft rejection after vaccination. We searched MEDLINE, Embase, and Cochrane CENTRAL until Jan 2024. Screening, data extraction, risk of bias and certainty of evidence assessment were assessed by two reviewers.

**Figure 2:**
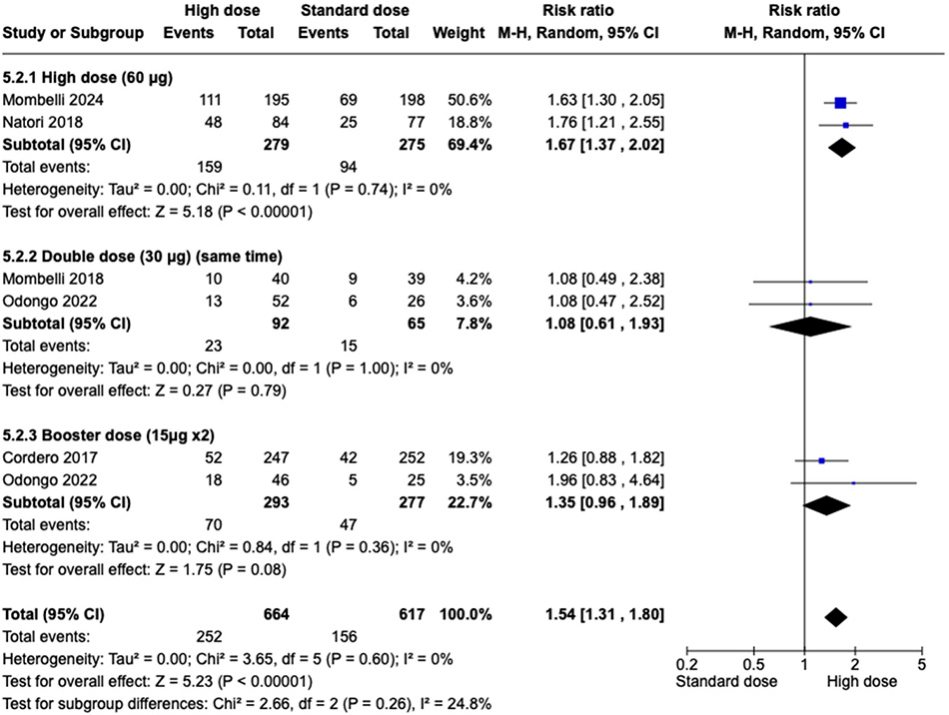
Seroconversion rate in Influenza A (H3N2) (SC H3N2) Forest plot

**Results:**

Pooling data from the 5 studies (1281 patients) shows moderate quality of evidence in the SCR towards using HD influenza vaccine in H1N1 type and high-quality evidence for H3N2 type . (RR 1.87 (1.25 to 2.79), RR 1.54 [1.31 to 1.80]). Analysis of five studies indicated that HD influenza vaccine results in no difference in graft rejection. (RR 0.73 [0.37 to 1.45]). High quality evidence showed that HD influenza vaccine reduce serious adverse events in SOT patients. (RR 0.71 [0.52 to 0.96]). Data from three studies shows HD influenza vaccine may results in little to no difference in confirmed clinically influenza. (low certainty of evidence) (RR 1.16 [0.58 to 2.29]).

**Figure 3:**
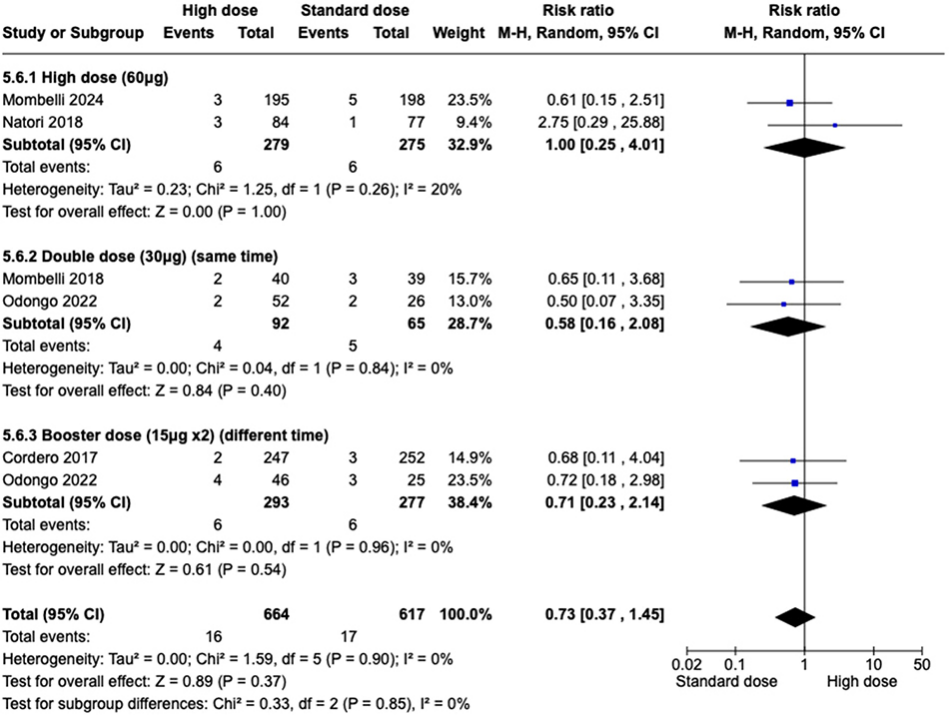
Graft rejection (GR) Forest plot

**Conclusion:**

Our systematic review indicates that HD influenza vaccine probably increases SCR in H1N1 and increases SCR in H3N2.HD vaccine, also may not increase graft rejection and reduces serious adverse events in SOT patients.

GRADE Summary of Findings (SoF) Table

**Figure:**
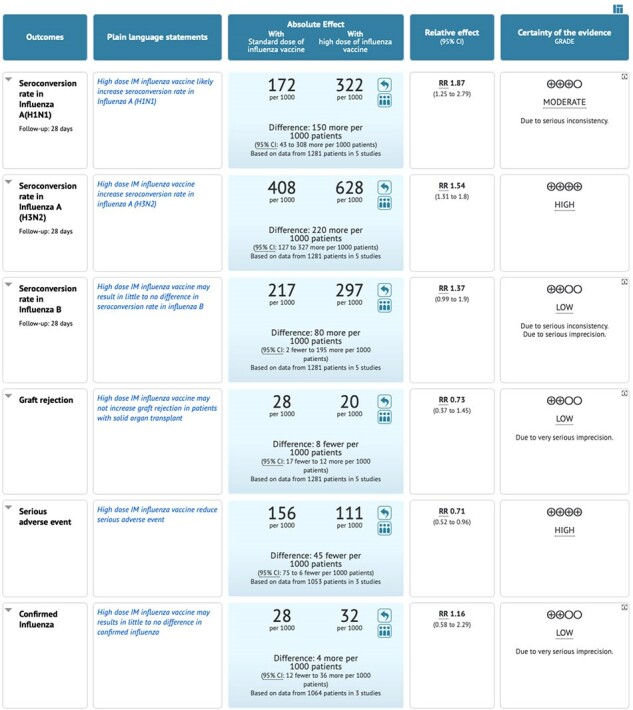

**Disclosures:**

**Reza Rahimi Shahmirzadi, MD**, Pfizer: Grant/Research Support **Meisam Abdar Esfahani, MD**, Pfizer: Grant/Research Support **Michael Silverman, MD, FRCP, FACP, AAHIVMed**, Pfizer: Grant/Research Support

